# Conventional small-bowel capsule endoscopy reading vs proprietary artificial intelligence auxiliary systems: Systematic review and meta-analysis

**DOI:** 10.1055/a-2544-2863

**Published:** 2025-03-14

**Authors:** Pablo Cortegoso Valdivia, Stefano Fantasia, Stefano Kayali, Ulrik Deding, Noemi Gualandi, Mauro Manno, Ervin Toth, Xavier Dray, Shiming Yang, Anastasios Koulaouzidis

**Affiliations:** 1 18630 Gastroenterology and Endoscopy Unit, University Hospital of Parma, Parma, Italy; 2 6174 Department of Clinical Research, University of Southern Denmark, Odense, Denmark; 3 11286 Department of Surgery, Odense University Hospital, Svendborg, Denmark; 4 18067 Gastroenterology and Digestive Endoscopy Unit, AUSL Modena, Carpi, Italy; 5 Department of Gastroenterology, Skåne University Hospital, Lund University, Malmoe, Sweden; 6 27063 Center for Digestive Endoscopy, Saint Antoine Hospital, APHP, Sorbonne Université, Paris, France; 7 6174 Faculty of Health Sciences, University of Southern Denmark, Odense, Denmark; 8 12525 Department of Gastroenterology, The Second Affiliated Hospital, Third Military Medical University, Chongqing, China; 9 53172 Department of Medicine, Svendborg Sygehus, Svendborg, Denmark; 10 11286 Surgical Research Unit, Odense University Hospital, Odense, Denmark; 11 37805 Department of Gastroenterology, Pomeranian Medical University in Szczecin, Szczecin, Poland

**Keywords:** Endoscopy Small Bowel, Capsule endoscopy, Small bowel endoscopy, Artificial Intelligence

## Abstract

**Background and study aims:**

Small-bowel capsule endoscopy (SBCE) is the gold standard for diagnosing small bowel (SB) pathologies, but its time-consuming nature and potential for human error make it challenging. Several proprietary artificial intelligence (AI) auxiliary systems based on convolutional neural networks (CNNs) that are integrated into SBCE reading platforms are available on the market and offer the opportunity to improve lesion detection and reduce reading times. This meta-analysis aimed to evaluate performance of proprietary AI auxiliary platforms in SBCE compared with conventional, human-only reading.

**Methods:**

A systematic literature search was conducted to identify studies comparing AI-assisted SBCE readings with conventional readings by gastroenterologists. Performance measures such as accuracy, sensitivity, specificity, and reading times were extracted and analyzed. Methodological transparency was assessed using the Methodological Index for Non-randomized Studies (MINORS) assessment tool.

**Results:**

Of 669 identified studies, 104 met the inclusion criteria and six were included in the analysis. Quality assessment revealed high methodological transparency for all included studies. Pooled analysis showed that AI-assisted reading achieved significantly higher sensitivity and comparable specificity to conventional reading, with a higher log diagnostic odds ratio and no substantial heterogeneity. In addition, AI integration substantially reduced reading times, with a mean decrease of 12-fold compared with conventional reading.

**Conclusions:**

AI-assisted SBCE reading outperforms conventional human review in terms of detection accuracy and sensitivity, remarkably reducing reading times. AI in this setting could be a game-changer in reducing endoscopy service workload and supporting novice reader training.

## Introduction

Since the introduction of small-bowel capsule endoscopy (SBCE) about two decades ago, indications for its use in clinical practice have been gradually implemented, and it has been accepted as the gold standard diagnostic tool for investigating small bowel (SB) pathology 1 . SBCE reading is a time-consuming task requiring high concentration levels, making it prone to human-related performance errors 2 . Consequently, SBCE has become a fertile ground for application of artificial intelligence (AI) algorithms 3 , which can provide automated detection (and possibly characterization) of lesions and reduce reading times while maintaining elevated performance measures (e.g., sensitivity and specificity). In addition, AI may play a role in training settings, enabling beginners to perform comparable (or even superior) to experienced readers 4 5 .

Technological advancement in recent decades has enabled integration of various machine learning (ML) models in medical devices 3 , especially after introduction of “deep learning”: the latter refers to a subtype of ML characterized by deep neural networks (DNNs), whose structure consists of several neuronal layers. Specifically, convolutional neural networks (CNNs) are DNN structures widely used for medical image analysis 6 . Training in ML occurs in a supervised or unsupervised manner, employing algorithms to adjust model parameters for optimal performance iteratively. Supervised learning relies on ground truth data (training set) to train ML systems, using these data as a benchmark for accuracy. Once trained, ML systems can make informed decisions and automatically extract image features. CNN and recurrent neural networks (RNNs) are the most advanced deep learning models applied to SBCE 7 .

In 2019, Ding et al. published the first report of a proprietary deep CNN algorithm integrated into one of the commercially available SBCE systems 8 . The CNN-based auxiliary model identified abnormalities with higher sensitivity levels and significantly shorter reading times than conventional analysis by experienced gastroenterologists. To date, NaviCam (AnX Robotica, Plano, Texas, United States) and OMOM (Jinshan Science & Technology, Chongqing, China) are the only capsule endoscopy systems to incorporate proprietary AI models, respectively named ProScan and SmartScan, both capable of selecting frames of interest within a video sequence, and regions of interest within these selected frames with SB abnormalities ( https://www.anxrobotics.com/products/navicam-sb-capsule-system/ ; https://www.jinshangroup.com/product/omom-hd-capsule-endoscopy-camera/ ). Because other manufacturers are working on similar solutions, many published papers on integrated CNNs exist today. Considering the large number of practical, non-branded, “home-grown” AI models for SBCE proposed by various authors 9 , the literature lacks a comprehensive review of the available, marketed, proprietary AI platforms in this specific setting. Consequently, this meta-analysis aimed to provide an up-to-date review of current performance of AI auxiliary reading platforms in SBCE compared with conventional (human-only) reading.

## Methods

### Data sources and search strategy

We conducted a systematic literature search in PubMed to identify all relevant studies in which the performance of proprietary AI software in detecting lesions was directly compared with standard reading by physicians. The primary outcome was evaluation of performance measures of both conventional and AI-assisted reading; the secondary outcome was assessing reduction in SBCE reading time using AI auxiliary platforms compared with conventional human reading. The last literature search was performed on July 18, 2024. A manual review of the reference list of included studies followed the electronic search. The complete search string is available in Supplementary Table 1.

### Inclusion and exclusion criteria

Inclusion criteria were: 1) full-text articles; 2) articles reporting performance measures of both conventional and proprietary AI-assisted reading in lesion detection; and 3) articles in the English language. Exclusion criteria were article types such as reviews/systematic reviews, editorials/perspectives/opinion pieces, individual case reports, letters to the editor, and commentaries.

### Screening of references

After excluding duplicates, three authors independently screened references (P.C.V., S.F., S.K.). Each author screened two-thirds of the references (title and abstract) according to the inclusion and exclusion criteria. In case of discrepancy, an article was included for full-text evaluation. This approach was repeated on included references with three authors' assessment of the full text (P.C.V., S.F., S.K.). In case of discrepancy in the full-text evaluation, the third author would also evaluate the reference, and a consensus discussion among all three would determine the outcome.

### Data extraction

Data were extracted following the Preferred Reporting Items for Systematic Reviews and Meta-Analyses (PRISMA) 15 . We extracted data on the SBCE model, type of AI auxiliary platform, number of patients and images in both the training and validation phases, per-patient and per-lesion analysis performance measures in conventional and AI-assisted reading, number of analyzed images, and reading times. Only data regarding the SB were extracted in studies assessing segments other than SB (e.g., stomach).

### Study assessment and risk of bias

The included studies underwent assessment of methodological transparency by two independent reviewers (P.C.V., S.F.) using the Methodological Index for Non-randomized Studies (MINORS) assessment tool 16 . Studies achieving over two-thirds of the maximum achievable score (24 for comparative studies) were considered highly transparent in methodology.

### Statistics

Statistics reported from each included study were used to visualize test performance of conventional and AI-assisted SBCE reading in crosshair and forest plots, including sensitivity, specificity, and false-positive rates. The number of true positives, true negatives, false positives, and false negatives from each included study was stratified by conventional and AI-assisted SBCE readings, either extracted directly from the studies or deduced from the available counts of total patient videos, total positive patient videos, and per-patient sensitivity. These quantities were used as input to calculate individual and pooled diagnostic odds ratios (ORs) as a comparative measure of test performance between conventional and AI-assisted SBCE readings. A random effects model was employed because no assumption of a common true effect size across the included studies could be made. In cases where input cells equaled zero, 0.5 was used for continuity corrections as by default in the utilized mada package in R. To ease reading and interpretation of the diagnostic ORs, we reported and visualized the log diagnostic ORs by forest plots including the pooled estimates, stratified by conventional and AI-assisted reading. The Cochranes Q and Higgins I ^2^ were employed to investigate the degree of heterogeneity between the included studies 17 18 19 .

## Results

**Fig. 1:**
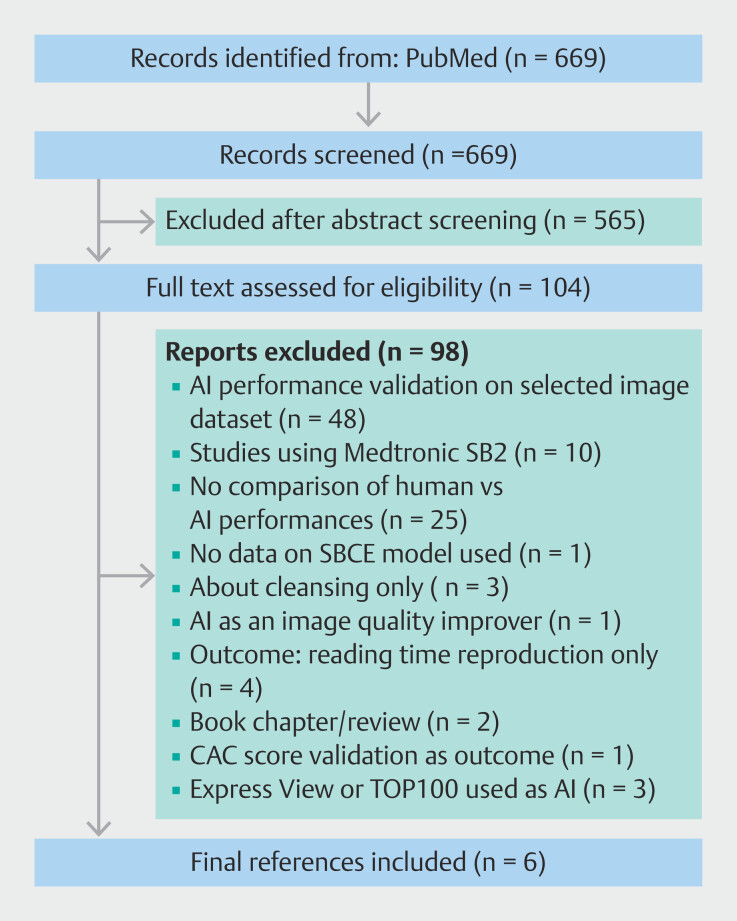
Flow diagram of the study. AI, artificial intelligence; CAC, computed assessment of cleansing; SBCE, small-bowel capsule endoscopy.

Overall, 669 references were identified in the initial search. Abstract screening excluded 565 records, leaving 104 references for full-text reading. Six studies (n = 6) were eventually included 8 10 11 12 13 14 , all of which reported comparisons on conventional and AI-assisted reading performance measures, enabling them to be included in pooled estimates ( Fig. 1 ). MINORS scores ranged from 16 to 24, highlighting high methodological transparency in all included studies ( Table 1 ). Validation procedures for each included study are depicted in Table 2 .

**Table:** **Table 1** Characteristics of included studies.

Study (year) [ref]	Study type	Software	AI model	Type of lesion	Training data		Validation data		MINORS (0–24)
					Patients (n)	Images (n)	Videos (n)	Images (n)	
Ding Z. (2019) 8	Multicenter Retrospective	NaviCam ESview	CNN (ProScan)	Any	1970	158,235	5000	113,268,334	17
Xie X. (2022) 10	Multicenter Retrospective	OMOM VUE smart	CNN (YOLO) (Smart Scan)	Any	2927	757,770	2898	146,956,145	18
Ding Z. (2023) 11	Multicenter Retrospective	NaviCam ESView	CNN + CRNN (ProScan)	Any	2565	280,426	240	5,741,518	16
O’Hara FJ. (2023) 12	Unicenter Retrospective	OMOM VUE smart	CNN (YOLO) (Smart Scan)	Any	NR	NR	39	NR	19
Spada C. (2024) 13	Multicenter Prospective	NaviCam ESView	CNN (ProScan)	Bleeding lesions	NR	NR	133	NR	24
Xie X. (2024) 14	Multicenter Retrospective	OMOM VUE smart	CNN (YOLO) (Smart Scan)	Any	1069	40,508	342	NR	18
AI, artificial intelligence; CNN, convolutional neural network; MINORS, Methodological Index for Non-randomized Studies; NR, not reported; YOLO, (you only look once) algorithm.									

**Table:** **Table 2** Validation procedures for included studies.

Study (year) [ref]	Validation procedure
Ding Z. (2019) 8	5000 videos were distributed to 20 expert gastroenterologists (250 each) for full conventional reading. Abnormal images detected by AI-assisted reading were revied manually by the same readers and a diagnosis was made. In case of discrepancy (conventional vs. AI-assisted), a final diagnosis was made after consensus among all 20 gastroenterologists. Final consensus diagnosis was considered the gold standard.
Xie X. (2022) 10	Stage 1: 2898 videos were distributed to 8 experienced gastroenterologists [>200 cases/year] (about 362 each) for full conventional reading. Stage 2 (after 6 months): AI-assisted reading by the same gastroenterologists. Stage 3 (after 3 months): 3 expert readers [>800 cases/year] provided adjudication on discordant cases. The combined agreed comparator was formed by concordant findings (stage 1&2) and discordant findings adjudicated by the group of expert readers (stage 3).
Ding Z. (2023) 11	240 videos were distributed to expert gastroenterologists for full conventional reading. Abnormal images detected by AI-assisted reading were reviewed manually by the same readers and a diagnosis was made. In case of discrepancy (conventional vs. AI-assisted), a final diagnosis was made after consensus among all 20 gastroenterologists. Final consensus diagnosis was considered the gold standard.
O’Hara FJ. (2023) 12	40 videos were distributed to 2 experienced gastroenterologists for full conventional reading - results were revied by an expert group (3 experienced [50–100 cases/year] and 2 expert [> 200 cases/year for > 15 years] gastroenterologists). AI-assisted reading was performed by same readers 3 months later, and each image selected by the algorithm was evaluated for findings.
Spada C. (2024) 13	Phase 1: investigators performed full conventional reading of videos at the site of patient’s enrolment (133 videos). Phase 2: after anonymization, videos were randomly reallocated to an external center for a second, AI-assisted reading. Phase 3: a board of 5 experts (> 500 cases) reviewed all videos to compare the results and to evaluate the match of findings of phases 1&2. In case of discrepancy, the board consensus reassessment was considered the gold standard.
Xie X. (2024) 14	Stage 1: 342 videos were distributed to experienced gastroenterologists [> 200 cases/year for 10 years] (342 each) for full conventional reading. Stage 2 (after 5 months): AI-assisted reading by the same gastroenterologists. Stage 3: 3 senior readers [> 300 cases/year for 15 years] provided adjudication on discordant cases. The combination of concordant findings (stages 1&2) and discordant findings adjudicated by the senior readers (stage 3) was considered the reference standard.
AI, artificial intelligence.	

### Performance comparison—per-patient analysis

**Fig. 2:**
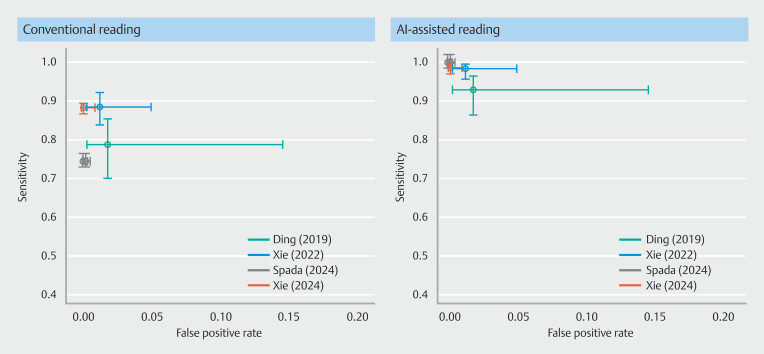
Crosshair plots. For each reading measure visualizing the sensitivity and false positivity rate estimates for each study included in the pooled per-patient analysis - the width of the whiskers indicates the study sample size (weight).

**Fig. 3:**
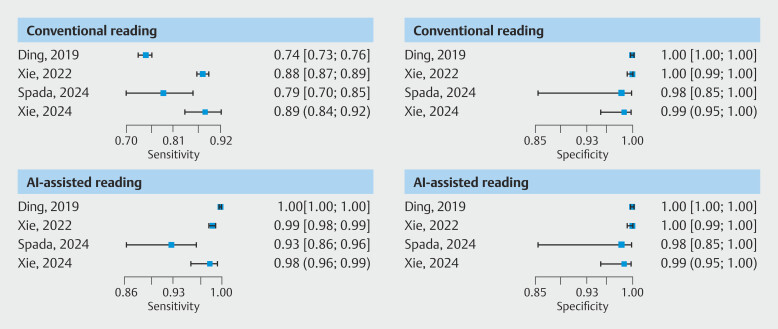
Forest plots of sensitivity and specificity for each study included in the pooled per-patient analysis, stratified by reading measure.

**Fig. 4:**
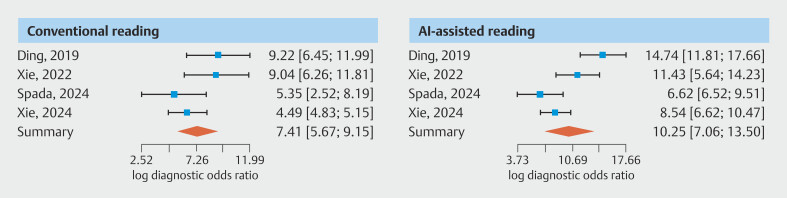
Pooled estimates of log diagnostic odds ratios for each reading measure (per-patient analysis).

The number of true positives, true negatives, false positives, and false negatives stratified by reading measure is reported for each study in Table 3 . Overall per-patient performance values were not reported in two studies 11 12 . The false-positive rate appears to be comparable between conventional and AI-assisted reading ( Fig. 2 ), whereas the sensitivity is markedly improved with AI reading compared with conventional reading. Sensitivity values in AI-assisted reading are 1.0, 0.99, 0.93, and 0.98, respectively, compared with 0.75, 0.88, 0.79, and 0.89 with conventional reading. Notably, no difference was found in specificity, because its values and related confidence intervals (CI) were identical ( Fig. 3 ). Pooled estimates of the log diagnostic OR were 7.4 (CI 95% 5.7–9.2) for conventional reading and 10.3 (CI 95% 7.1–13.5) for AI-assisted reading. Prediction intervals were 4.95 to 9.87 for conventional reading and 4.40 to 16.16 for AI-assisted reading. For all four studies included in the per-patient pooled analysis 8 10 13 14 , the log diagnostic OR estimate was higher with AI-assisted reading than with conventional reading, with overlapping CIs ( Fig. 4 ). No substantial heterogeneity was observed in either the pooled analysis for conventional reading (Cochran's Q: 3.179 (3 df, *P* = 0.365), Higgins' I ^2^ : 5.6 %) or AI-assisted reading (Cochran's Q: 3.394 (3 df, *P* = 0.335), Higgins' I ^2^ : 11.6%.

**Table:** **Table 3** Quantitative input used to estimate diagnostic odds ratios for pooled meta-analysis (per-patient analysis).

Study	True positives		False positives		False negatives		True negatives	
	Conventional	AI	Conventional	AI	Conventional	AI	Conventional	AI
Ding, 2019	2443	3272	0	0	833	4	1724	1724
Xie, 2022	2048	2298	0	0	278	28	572	572
Spada, 2024	83	98	0	0	22	7	28	28
Xie, 2024	191	212	1	0	24	3	126	126
AI, artificial intelligence.								

### Performance comparison—per-lesion analysis

Five studies reported per-lesion performance indicators. Because the definition of pathological findings differed significantly among the included studies, we only considered overall values. Regarding accuracy and sensitivity, AI-assisted reading achieved statistically significantly higher results than conventional reading, as demonstrated by the *P* values ( Table 4 ). Regarding specificity, AI-assisted reading obtained 100% and 97.1%, respectively 8 11 , compared with 100% for conventional reading (expert consensus was the comparator).

**Table:** **Table 4** Overall performance values (per-lesion analysis).

Study	Performance value	Conventional	AI-assisted	*P* value
Ding, 2019	Accuracy	54.57%	70.91%	NR
	Sensitivity	76.89%	99.90%	< 0.0001
	Specificity	100%	100%	> 0.99
Xie, 2022	Accuracy	76.10%	95.90%	< 0.001
	Sensitivity	NR	NR	–
	Specificity	NR	NR	–
Ding, 2023	Accuracy	96.6%	97.9%	NR
	Sensitivity	91.1%	99.2%	< 0.0125
	Specificity	100%	97.1%	< 0.0125
O'Hara, 2023	Accuracy	NR	NR	–
	Sensitivity	86.2%	98.1%	< 0.001
	Specificity	NR	NR	–
Xie, 2024	Accuracy	84.79%	97.24%	< 0.001
	Sensitivity	NR	NR	–
	Specificity	NR	NR	-–
AI, artificial intelligence; NR, not reported.				

### Image and reading time reduction

**Fig. 5:**
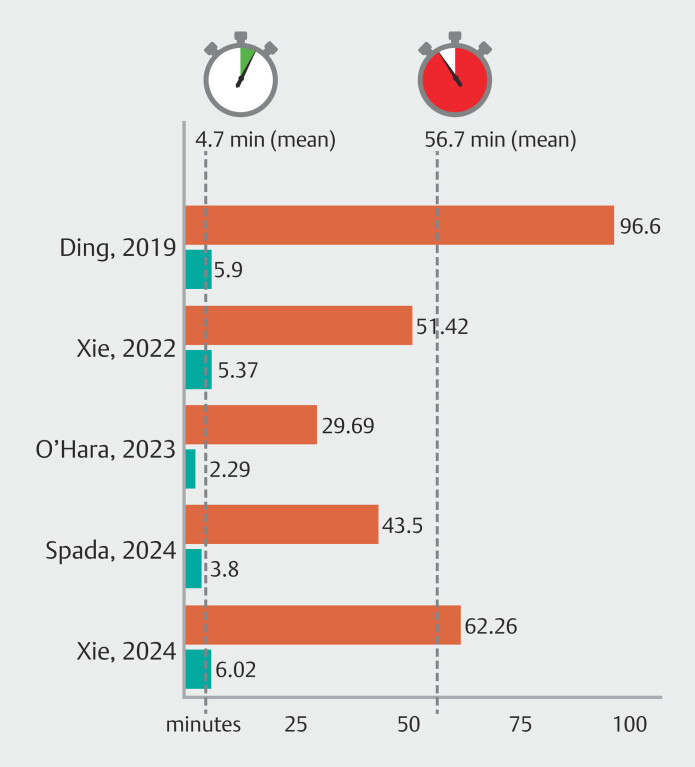
Reading times. Red color: conventional reading; green color: AI-assisted reading.

AI software provided a net reduction in number of images to review. In detail, the decrease in mean number of images from the four (n = 4) studies providing the data was 39-fold 8 , 36-fold 10 , 24-fold 13 , and 51-fold 14 . This aspect was then translated to reduced reading time: AI-assisted reading required a mean time of 4.7 minutes, whereas conventional reading required 56.7 minutes ( Fig. 5 ).

## Discussion

One of the primary concerns for gastroenterologists when analyzing images from lengthy SBCE videos is risk of missing lesions, because these may appear in only a few of the tens of thousands of frames that comprise the entire video sequence. This aspect is burdened by the time required to review a recording, which may decrease with experience but is admittedly associated with the potential for missing lesions 20 . A seminal paper by Beg et al. showed that reader accuracy declines after just one SBCE reading 2 . Therefore, the time required to report each exam is crucial to ensure reading quality, especially when multiple exams are reviewed consecutively. In addition, disappointing results in terms of interobserver/intraobserver agreement and detection rate of significant findings, regardless of SBCE reader experience, have been underscored in previous works 21 . A paper by Rondonotti et al. showed that dedicated training programs did not significantly increase performance of readers with different levels of experience 22 .

The results of our study highlight that AI-assisted reading provides superior diagnostic performance in terms of accuracy and sensitivity in both per-lesion and per-patient analyses across all included studies, showing a higher diagnostic OR for AI-assisted reading compared with conventional reading (10.3 versus 7.4) despite overlapping CIs. This finding reassures clinicians about reduced risk of missing pathology. It confirms that AI software fulfills its intended purpose: allowing the reader to focus solely on the most ambiguous lesions by filtering out the “noise” of thousands of negative images. In this context, our results support the role of AI-assisted reading in paving a new era of reduced reviewing times. The mean SBCE reading time achieved with AI assistance was 12 times shorter than that required for conventional reporting (4.7 versus 56.7 minutes, respectively). This opens new potential perspectives, not only in terms of performance quality but also in terms of healthcare costs. Future studies should be conducted to explore this aspect further.

Interestingly, AI auxiliary platforms may also play a role in closing the gap between novice and expert readers in SBCE reading. According to the results of Ding et al. and Xie et al. 11 14 , incremented sensitivity for SB lesions of AI-assisted junior readers even surpassed that of experts in conventional reading mode (99.2% and 96.7% versus 91.1% and 88.8%, respectively). Compared with conventional reading, in the study by Ding et al., the same novice readers obtained a reduction of 33.3% in missed diagnosis rate (34.1% conventional, 0.8% AI-assisted 11 . These data underscore the clinical importance of AI auxiliary reading in training by improving efficiency and work performance.

Our study has several limitations, primarily related to characteristics of the included studies. First, only six studies were included, five of which were retrospective. Second, in the per-patient analysis, aggregated data included only four of six studies, potentially reducing the strength of the pooled analysis. Third, data pooling was impossible in the per-lesion analysis due to heterogeneity in the definition of pathological findings among the studies; therefore, only overall lesion values were compared with their respective conventional reading counterparts. Fourth, an intrinsic clinical limitation concerns the comparator. Because all studies considered expert consensus/board the ground truth in cases of uncertainty, one must consider inherent agreement variations among observers (even if experts). On the other hand, deep enteroscopy is the only third-party method capable of addressing this limitation, and it cannot be performed on all patients for obvious ethical and technical reasons.

However, several strengths of this work should be highlighted. To our knowledge, this is the first systematic review with a pooled analysis addressing the role of proprietary AI models in diagnostic workup of SBCE published in the literature. The decision to focus exclusively on proprietary software may be subject to criticism and praise. However, given our aim to provide a practical perspective for clinicians, concentrating on available AI systems—readily reproducible and globally applicable—represents a key strength of our study. Furthermore, our findings are bolstered by high reliability due to the low heterogeneity level observed in the pooled analyses.

## Conclusions

In conclusion, compared with conventional video review, AI-assisted reading shows superior diagnostic performance by increasing detection accuracy and sensitivity, remarkably reducing reading times. Nevertheless, caution is advised: auxiliary AI tools cannot yet fully replace expert human reading, especially due to ethical aspects. At this stage, our findings advocate for widespread adoption of AI auxiliary software to assist clinicians in SBCE reading, easing the workload of endoscopy services and exerting a supportive role in the training curve for novice readers.

## Supplementary Material

Supplementary Material Zusatzmaterial
