# Supplementary material for: Conventional small-bowel capsule endoscopy reading vs proprietary artificial intelligence auxiliary systems: Systematic review and meta-analysis
Source: Endosc Int Open. 2025 Mar 14;13:a25442863. doi: 10.1055/a-2544-2863 (PMC11922306; doi:10.1055/a-2544-2863)
Supplement: Supplementary file 1 — Supplementary Material [file 10-1055-a-2544-2863_25482030.pdf]

Supplementary Table 1 Systematic literature search.

PubMed
(capsule endoscopy OR capsule endoscope OR capsule OR videocapsule OR pillcam OR panenteric) AND (small intestine OR small intestine disease OR small intestine tumor OR small bowel) AND (artificial intelligence OR machine learning OR software OR algorithm OR automation OR computer analysis OR computer assisted diagnosis OR image processing OR software OR algorithm OR automat OR support-vector OR svm OR hybrid OR neural-network OR autonom)

Endosc Int Open | DOI 10.1055/a-2544-2863 | © The Author(s).
